# Prevalence and factors associated with adolescent idiopathic scoliosis in Shanghai, China: an artificial intelligence-assisted screening approach

**DOI:** 10.3389/fpubh.2026.1832421

**Published:** 2026-06-24

**Authors:** Yuanyuan Cao, Fangfang Li, Yong Zhang, Wenwen Gu, Xinfeng Zheng, Meng Li, Xu Liu, Mengjia Feng, Xilin Yang, Yulu Zhang, Shu Xie, Lijuan Yu, Weibing Wu, Jun Xia

**Affiliations:** 1 School of Exercise and Health, Shanghai University of Sport, Shanghai, China; 2 Education Bureau of Baoshan District, Shanghai, China; 3 Department of Spine Surgery, Xinhua Hospital Affiliated to Shanghai Jiaotong University School of Medicine, Shanghai, China; 4 Dongciyuan Intelligent Technology Co., Ltd., Shanghai, China

**Keywords:** 3D camera, adolescent idiopathic scoliosis, AI-assisted screening, machine learning, prevalence, risk factors

## Abstract

**Objective:**

To investigate the prevalence of adolescent idiopathic scoliosis (AIS) and major modifiable factors associated with AIS among school students in Shanghai, China, and assess the feasibility and validated accuracy of AI-assisted 3D screening in school health management.

**Methods:**

A multistage stratified cluster sampling design was employed in this cross-sectional study, conducted from July 2024 to June 2025. A structured-light 3D back-scanning AI system was used to screen 5,026 students aged 9–15 years from six schools in five districts of Shanghai. Physical fitness tests and questionnaires were used to collect data on body morphology, posture, physical activity, and lifestyle. Standing full-spine radiography was used as the reference standard for AIS diagnosis, defined as Cobb angle ≥10°. Because radiographic verification was performed for all screen-positive students but only for a random subset of screen-negative students, inverse probability weighting was applied to account for partial verification.

**Results:**

Among the 5,026 screened students, 160 AIS cases were confirmed among 185 rescreen-positive students, and 2 additional AIS cases were identified among 300 randomly verified screen-negative students. The crude radiographically confirmed detection rate was 3.2% (162/5,026), and the IPW-adjusted estimated population prevalence was 3.8%. Thoracic curves and Cobb angles of 10°– < 20° were most common. Girls and middle school students had higher crude radiographic detection rates than boys and primary school students, respectively. Multivariable binary logistic regression identified female sex, improper sitting posture among girls, very low post-exercise fatigue among boys, and frequent one-sided exertion sports as factors associated with screening-detected AIS, whereas higher BMI, balanced walking posture, and satisfactory sleep quality showed protective associations. The AI-assisted structured-light screening workflow showed IPW-adjusted sensitivity of 0.832, specificity of 0.995, PPV of 0.865, NPV of 0.993, and overall accuracy of 0.989.

**Conclusion:**

AIS represents a non-negligible spinal health burden among school students in Shanghai. Most factors associated with AIS are modifiable. The AI-assisted structured-light screening workflow demonstrated acceptable diagnostic accuracy and promising feasibility for large-scale school-based health screening and management integration.

## 1 Introduction

Adolescent idiopathic scoliosis (AIS) is a three-dimensional spinal deformity of unknown etiology, typically occurring during periods of rapid growth in adolescence. The diagnosis is confirmed when the Cobb angle on full-spine radiographs is ≥10^°^ (1, 2). The global prevalence of AIS among adolescents ranges from 0.47 to 5.20% (3). In China, the average detection rate is approximately 2.8% (4), affecting more than 5 million children and adolescents, with an estimated 300,000 new cases annually (5). Because AIS is often asymptomatic in its early stages, failure to detect and intervene in time may lead to rapid progression during puberty, resulting in significant physical deformities, chronic pain (6), cardiopulmonary dysfunction (7), and psychological issues (8). Therefore, early identification of AIS and its potential signs is critical for mitigating long-term health burdens (9–11).

While school-based methods provide basic diagnostic capacity, they are limited by subjectivity, low efficiency for follow-up, and a lack of continuous, quantitative data (11–13). Recent advances in artificial intelligence (AI) have improved adolescent spinal health screening through three main approaches: first, image-based CNN algorithms for detecting abnormalities in radiographs or surface images (14–17); second, non-contact 3D reconstruction using structured-light or depth cameras to assess trunk symmetry via geometric or machine-learning models (18); and third, dynamic posture and movement analysis, such as gait monitoring or electromyography combined with deep learning for risk prediction (19). Among these, structured light depth cameras integrated with AI offer key advantages: non-contact, radiation-free, automated, and quantifiable results. These technologies greatly enhance efficiency and support early, large-scale AIS detection and intervention. Full-spine radiographs can then be performed in screening-positive students to confirm the diagnosis while minimizing unnecessary radiation exposure (20, 21).

Amid increasing urbanization, adolescents in megacities face heightened risks of postural imbalance and spinal curvature due to academic stress, physical inactivity, and frequent use of electronic devices (22, 23). Multiple studies have reported detection rates of spinal curvature abnormalities in urban regions significantly higher than national averages—for example, 8.2% in Guangzhou (24), 5.7% in Shenzhen (25), 1.4–2.2% in Singapore (26), 3.9% in certain Korean cities (27), 2–4% in the United States (9), up to 5% in Germany (28), and 3.9% in Australia (29). However, systematic research integrating intelligent screening technologies with epidemiological assessments in megacity settings remains scarce. This study focuses on Shanghai, a representative Chinese megacity, using a hybrid screening approach that combines traditional assessments with an AI-based structured light system. Through physical measurements and questionnaires, we assess the detection rate, sex- and age-related differences, and factors associated with AIS in primary and secondary school students. While AI and structured-light technologies have been explored in smaller studies, large-scale implementation in real-world school health systems remains limited. This study pioneers the large-scale application of AI-assisted structured-light screening in the school health system of Shanghai, integrating postural assessment with comprehensive analysis of morphology, activity, and lifestyle factors, and validating the screening results with radiographs, thereby providing new evidence and a practical pathway for AIS screening and health management in school settings.

## 2 Methods

### 2.1 Study design and participants

A multistage stratified cluster sampling design was employed in this cross-sectional study, conducted from July 2024 to June 2025. Based on an urban–rural stratification framework, five administrative districts of Shanghai were selected. Within each district, students from grades 4 through 7 (ages 9–15 years) were sampled at the class level across six schools, including both primary and junior secondary schools.

The inclusion criteria were as follows: (1) age between 9 and 15 years, regardless of sex; (2) in generally good health, without severe medical or surgical conditions; (3) no history of corrective surgery for spinal deformities; and (4) ability to comprehend the study procedures and provision of written informed consent by the participant and/or legal guardian. The exclusion criteria included: (1) current diagnosis and treatment for other spinal diseases; (2) presence of severe visual, auditory, or communication impairments; (3) significant comorbidities affecting spinal assessment or physical activity, such as cardiopulmonary disorders or neuromusculoskeletal conditions; and (4) refusal or inability of the student or guardian to cooperate with screening procedures or understand the study requirements.

Among 5,310 students initially recruited, 284 were excluded because they lacked valid questionnaire or screening data, leaving 5,026 students in the final screening cohort. Among the participants, 2,718 were male and 2,308 were female; 1,228 students were in grades 4–5 (primary school), and 3,798 students were in grades 6–7 (preparatory to junior high school).

**Figure 1:**
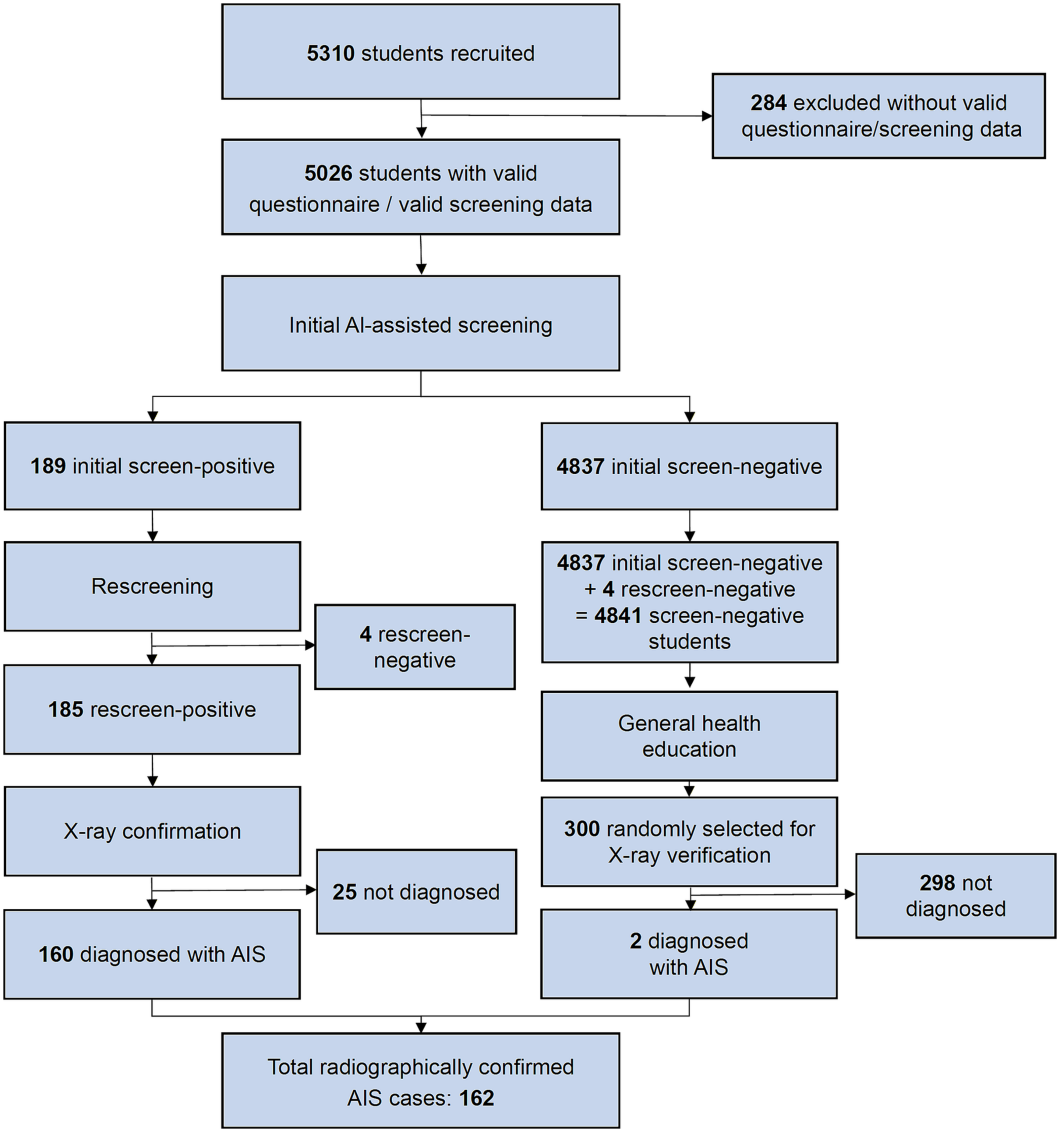
Flow diagram of data collection.

This study was approved by the Institutional Review Board of Shanghai University of Sport (Approval No. 102772024RT110). Written informed consent was obtained from all participants’ parents or legal guardians for both the spinal screening and questionnaire components. The flow diagram of data collection is shown in Figure 1.

### 2.2 Study procedures

#### 2.2.1 Screening for adolescent idiopathic scoliosis

To assess the prevalence of AIS among adolescents in Shanghai, China, a structured screening and diagnostic protocol for AIS was implemented. Eligible participants, as determined by the aforementioned inclusion and exclusion criteria, underwent a screening process adapted from internationally recognized guidelines published by the Scoliosis Research Society (SRS) (30) and the American Academy of Pediatrics (AAP) (31), as well as the national standards outlined in the Screening Guidelines for Abnormal Spinal Curvature in Children and Adolescents issued by the National Health Commission of China (32). In this study, a novel artificial intelligence (AI)-assisted system was integrated into the standard protocol, comprising three sequential phases: preliminary screening, rescreening, and clinical diagnosis.

(1) Preliminary screening

This study employed a dual-stage preliminary screening approach combining manual examination with AI-assisted 3D imaging, enabling both clinical judgment and objective surface measurements to identify potential spinal asymmetry.

Manual assessment included general physical examination, the Adam’s forward bend test, and postural correction and spinal mobility reassessment. The general examination evaluated the following indicators: (1) shoulder height asymmetry (≥1.5 cm); (2) scapular asymmetry at the inferior angle (≥1.5 cm); (3) lumbar bulge asymmetry; (4) iliac crest height discrepancy; and (5) deviation in the alignment of the spinous processes. The ≥1.5 cm threshold for shoulder height and scapular asymmetry was used as a pre-specified operational criterion for standardized field screening, rather than as a diagnostic criterion for AIS. The presence of any one of these findings was considered a positive result for component A. During the Adam’s forward bend test, the angle of trunk rotation (ATR) was measured using a scoliometer. An ATR ≥ 5° was defined as a positive result for component B. If either component A or B yielded a positive result, the participant underwent postural correction and spinal mobility reassessment. Participants whose ATR decreased to <5° after reassessment were classified as having postural abnormality rather than suspected structural scoliosis. Participants whose ATR remained ≥5° after reassessment were classified as preliminarily screen-positive based on manual assessment.

**Figure 2:**
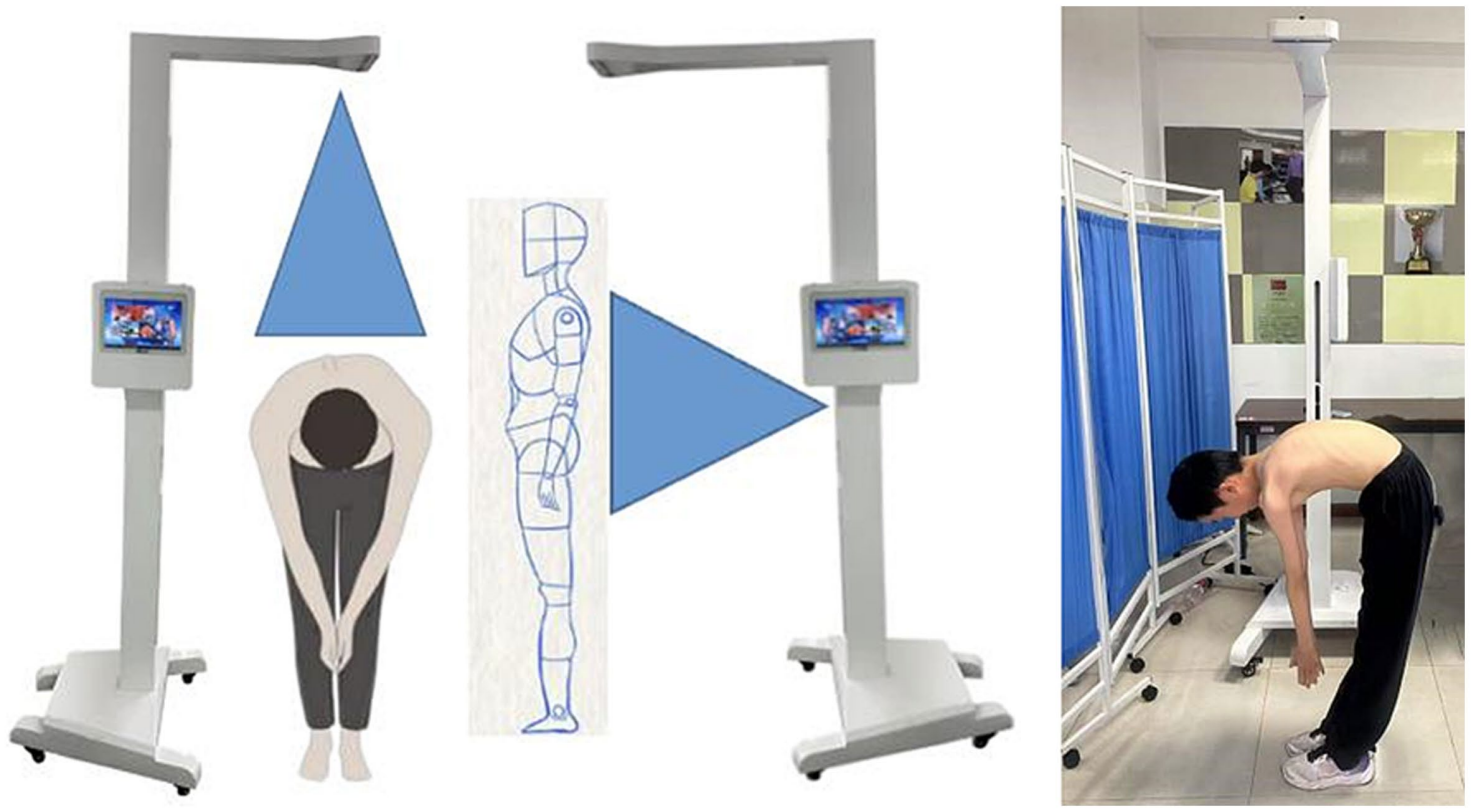
Schematic diagram of the AI-based spinal screening system.

**Figure 3:**
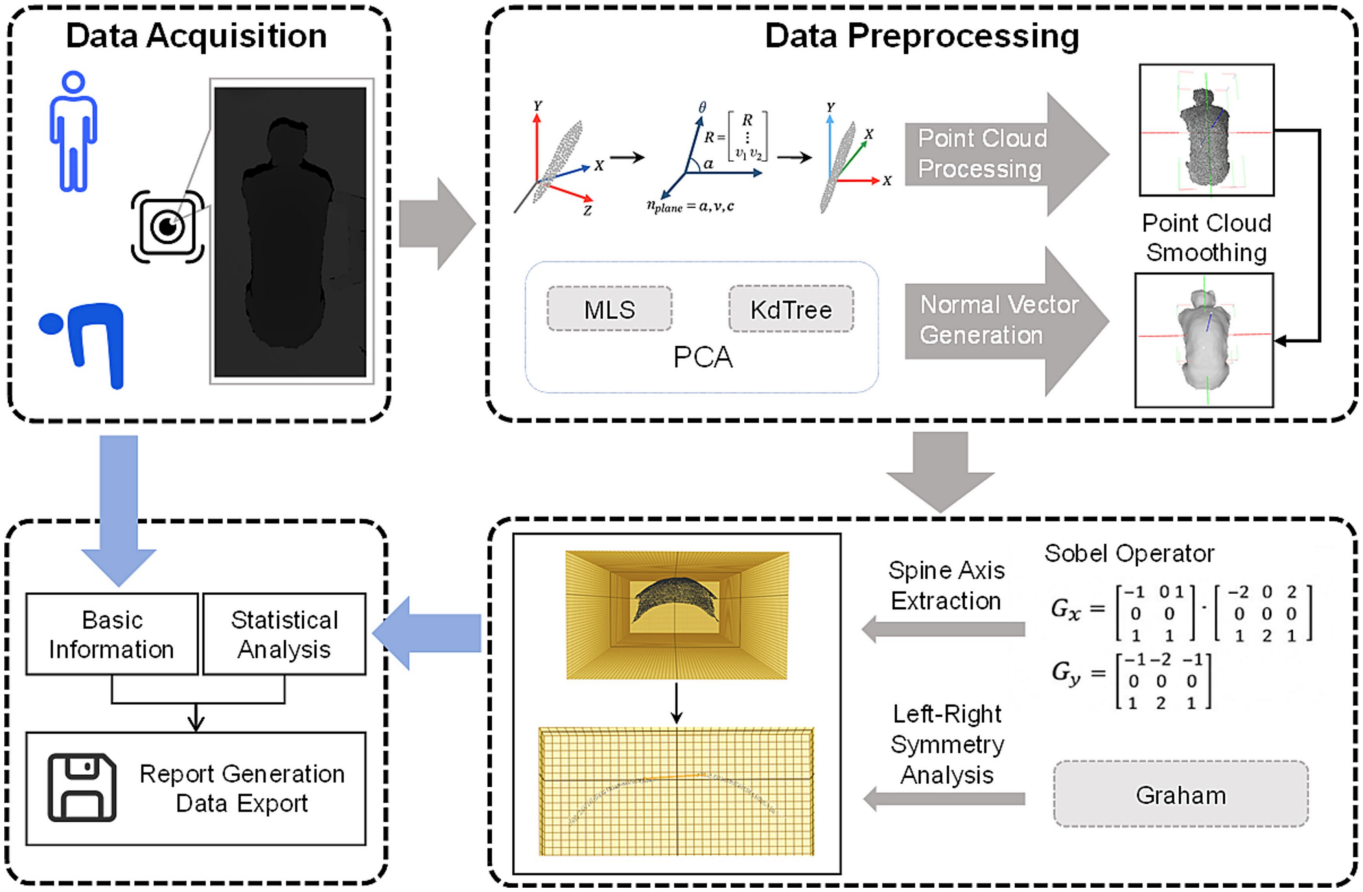
A deep perception-based, non-contact intelligent screening framework for scoliosis.

Following manual screening, all participants—including those with negative findings—underwent AI-assisted evaluation. A non-contact, AI-powered screening system employing structured light 3D depth imaging was used to acquire high-resolution dorsal surface data (Figure 2). Scans were performed under both natural standing posture and Adam’s forward bending posture. The system applied point cloud reconstruction and principal component analysis (PCA) to standardize postural orientation, and automatically identified key anatomical regions such as the scapular area, thoracic spine, and lumbar spine. It extracted quantitative features including surface height differentials, symmetry indices, and body surface deviation curves to model the spinal midline. The system computed ATR values for the thoracic, thoracolumbar, and lumbar segments, and generated quantitative reports with visual outputs to assist in localizing and grading the curvature (Figure 3). All school-based screenings were carried out by staff who had completed a standardized training on visual inspection, Adam’s forward bend testing, scoliometer use, and operation of the structured-light 3D system under manufacturer guidance.

(2) Rescreening

Participants who screened positive in the preliminary screening underwent a second round of screening using the same protocol as the initial phase, including repeated manual examination, postural correction and spinal mobility reassessment, and repeated AI-assisted structured-light assessment. Participants were classified as screen-negative only when both the manual assessment and the AI-assisted assessment were negative during rescreening. Participants were classified as rescreen-positive and referred for radiographic confirmation if the manual assessment remained positive, with ATR persisting at ≥5° after reassessment, or if the AI-assisted structured-light assessment remained positive.

Participants were categorized into five groups based on their angle of trunk rotation (ATR) and physical examination findings (33): No Curvature: No physical signs and ATR < 5^°^. Poor Posture: Presence of physical signs or ATR ≥ 5^°^, but ATR < 5^°^ after postural correction and spinal mobility reassessment. Grade I Abnormality: 5^°^ ≤ ATR < 7^°^. Grade II Abnormality: 7^°^ ≤ ATR < 10^°^. Grade III Abnormality: ATR ≥ 10^°^.

(3) Clinical diagnosis

Participants who screened positive in the rescreening phase were referred to designated hospitals for full-spine standing anteroposterior and lateral radiographs. Two board-certified radiologists independently reviewed the images and measured the Cobb angle. A diagnosis of AIS was confirmed when the Cobb angle was ≥10^°^.

(4) Evaluation of diagnostic performance of the AI-assisted screening workflow

Diagnostic performance was assessed for the integrated AI-assisted school-based screening workflow rather than for the AI algorithm alone. The workflow included manual examination, Adam’s forward bend test, scoliometer-based ATR measurement, AI-assisted structured-light 3D back scanning, rescreening, and radiographic confirmation. Participants who remained positive at rescreening according to either manual or AI-assisted assessment were classified as workflow-positive and referred for standing full-spine radiographs. A random sample of 300 workflow-negative students also underwent radiography to assess false-negative results. AIS was defined as a Cobb angle ≥10°. Sensitivity, specificity, positive predictive value, negative predictive value, and accuracy were calculated against radiographic confirmation, with inverse probability weighting applied to account for partial verification.

#### 2.2.2 Physical fitness and morphological assessment

To examine the associations between physical fitness, body morphology, and AIS among adolescents in Shanghai, China, several key physiological indicators were measured, including height, weight, handgrip strength, skinfold thickness, and body circumferences.

Height and weight were measured on-site by trained personnel using standardized devices of the same model. Participants were asked to remove their shoes and wear lightweight clothing during measurement. Height and weight were recorded with a precision of 0.1 cm and 0.1 kg, respectively. Body mass index (BMI) was calculated as weight divided by height squared (kg/m^2^).

Muscular strength was assessed through handgrip strength using an electronic dynamometer. Each participant stood in a relaxed upright position and completed two trials with each hand. The maximum value among the four attempts was recorded as the final result.

Body density was estimated using skinfold thickness measurements at three standard anatomical sites: the triceps, subscapular region, and abdomen. Measurements were obtained using a Harpenden skinfold caliper, with two readings per site averaged for analysis. Sex-specific equations developed by Jackson and Pollock were used to estimate body density, which served as an indirect indicator of body fat content and body composition (34).

Body circumference measurements included the chest, waist, and hips. Chest circumference was measured at both maximal inhalation and maximal exhalation to assess thoracic expansion and respiratory flexibility. Waist and hip circumferences were recorded at the narrowest and widest points, respectively, and the waist-to-hip ratio (WHR) was calculated as the ratio of waist circumference to hip circumference.

#### 2.2.3 Questionnaire survey

To explore the influence of physical activity, postural habits, and lifestyle factors on AIS among adolescents in Shanghai, China, two questionnaires were administered: *the Adolescent Lifestyle Survey and Evaluation Questionnaire* and the Chinese version of the *International Physical Activity Questionnaire–Short Form (IPAQ-SF)*.

**Figure 4:**
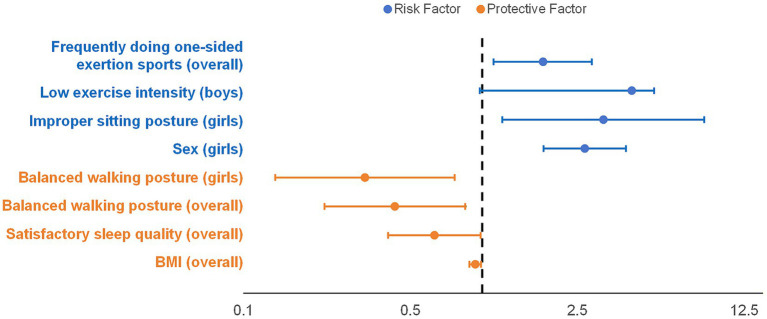
Significant factors associated with AIS and sex differences in adolescent idiopathic scoliosis.

*The Adolescent Lifestyle Survey and Evaluation Questionnaire* was developed in-house by the research team based on a comprehensive review of relevant literature and tailored to the context of primary and secondary school students in Shanghai. The questionnaire was finalized through expert consultation and iterative revisions involving specialists in spinal health, public health, and education. It consists of two sections with a total of 35 items. The first section contains 15 items focused on spinal health, including demographic characteristics (e.g., name, sex, age, grade), habitual postures (e.g., walking, sitting, standing, backpack weight, desk height), and spine-related behaviors (e.g., history of clinical visits, treatment, regular check-ups, and family history of spinal disorders). The second section comprises 20 items related to lifestyle, covering five domains: dietary habits, sleep quality, physical activity, health behaviors, and psychological stress. A pilot test of the questionnaire was conducted among a subsample of 120 students prior to formal data collection. Results indicated good internal consistency, with a Cronbach’s alpha of 0.80, and acceptable content validity (Figure 4).

The *IPAQ-SF* was used to assess physical activity during the previous 7 days, including time spent on vigorous activity, moderate activity, walking, and sedentary behavior. Energy expenditure for each activity was calculated using metabolic equivalent (MET) values, with assignments of 8.0 METs for vigorous activity, 4.0 METs for moderate activity, and 3.3 METs for walking. The total activity volume for each category was computed as the product of duration (minutes), frequency (days per week), and corresponding MET value. Overall physical activity level was expressed in MET·minutes per week. According to IPAQ scoring guidelines, participants were classified into three categories: low activity (<600 MET·min/week), moderate activity (600–3,000 MET·min/week), and high activity (>3,000 MET·min/week).

#### 2.2.4 Statistical analysis

All statistical analyses were performed using SPSS software, version 28.0 (IBM Corp). The Shapiro–Wilk test was used to assess the normality of continuous variables. Variables that followed a normal distribution were expressed as mean ± standard deviation and compared using independent-samples t tests. Categorical variables were presented as frequencies and percentages and compared using the chi-square test or Fisher’s exact test, as appropriate, whereas ordered categorical variables were analyzed using the Mann–Whitney *U* test. To identify factors associated with screening-detected AIS, multivariable binary logistic regression was conducted with AIS screening status as the dependent variable (AIS = 1; non-AIS = 0). Multivariable models were fitted using complete-case data. For prevalence estimation and diagnostic performance evaluation, because radiographs were obtained for all screen-positive students but only for a random sample of screen-negative students, inverse probability weighting (IPW) was applied to account for partial verification. Screen-positive students were assigned a weight of 1, and verified screen-negative students were assigned a weight of 4,841/300. The crude radiographic detection rate was defined as the unweighted proportion of radiographically confirmed AIS cases among all screened students, whereas the IPW-adjusted prevalence was calculated as the weighted number of AIS cases divided by the total number of screened students. The same weighting approach was used to obtain IPW-adjusted diagnostic performance estimates. Diagnostic performance of the AI-assisted school-based screening workflow against the radiographic reference standard was summarized using sensitivity, specificity, positive predictive value (PPV), negative predictive value (NPV), and overall accuracy. All statistical tests were two-sided, and a significance level of *α* = 0.05 was considered statistically significant.

## 3 Results

### 3.1 Screening outcomes and clinical characteristics of adolescent idiopathic scoliosis

#### 3.1.1 Screening outcomes and prevalence estimation

Among the 5,310 students initially recruited, 284 were excluded because they lacked valid questionnaire or screening data, leaving 5,026 students in the final screening cohort. Among the 5,026 students who participated in AIS screening, 189 (3.8%) tested positive at the initial screening, and 185 (3.7%) remained positive after rescreening. All 185 rescreen-positive students underwent radiographic confirmation, among whom 160 were diagnosed with AIS (Cobb angle ≥10°). In addition, among the 4,841 screen-negative students, 300 were randomly selected for radiographic verification, and 2 additional AIS cases were identified. Therefore, a total of 162 students were radiographically confirmed with AIS in the verification sample. The crude radiographically confirmed AIS detection rate was 3.2% (162/5,026). Because only a random subset of screen-negative students underwent radiography, this crude rate was not interpreted as the population prevalence. After IPW correction for partial verification, the estimated number of AIS cases was 192.3, and the IPW-adjusted estimated population prevalence was 3.8% (192.3/5,026).

By sex, 55 of 2,718 boys were diagnosed with AIS, corresponding to a crude radiographic detection rate of 2.0%, whereas 107 of 2,308 girls were diagnosed, corresponding to a crude radiographic detection rate of 4.6%; the crude detection rate was significantly higher in girls than in boys (*p* < 0.001). When stratified by school level, 25 of 1,228 primary school students and 137 of 3,798 middle school students were diagnosed with AIS, corresponding to crude radiographic detection rates of 2.0 and 3.6%, respectively (*p* = 0.01). Further stratification by grade showed crude radiographic detection rates of 1.4% in Grade 4, 2.7% in Grade 5, 3.9% in Grade 6, and 3.1% in Grade 7 (*p* = 0.011) (Table 1).

**Table 1:** Criteria for determining abnormal spinal curvature screening results.

Category		Screening result
No scoliosis		No abnormalities in general inspection and forward bend test, and ATR < 5°, determined as no scoliosis.
Postural abnormality		Abnormal general inspection or positive forward bend test or ATR ≥ 5°, but ATR < 5° after spinal mobility reassessment using a scoliometer.
Abnormal spinal curvature	Grade I	Abnormal general inspection or positive forward bend test or ATR ≥ 5°, and ATR is between 5° and 7° after spinal mobility reassessment using a scoliometer.
	Grade II	Abnormal general inspection or positive forward bend test or ATR ≥ 5°, and 7° ≤ ATR < 10° after spinal mobility reassessment using a scoliometer.
	Grade III	Abnormal general inspection or positive forward bend test or ATR ≥ 5°, and ATR ≥ 10° after spinal mobility reassessment using a scoliometer.

#### 3.1.2 Curve location and severity among radiographically confirmed AIS cases

In terms of curve location, thoracic curves were more common in boys (58.2%), followed by thoracolumbar (27.3%) and lumbar curves (12.7%). In girls, thoracic curves accounted for 42.1%, while lumbar and thoracolumbar curves accounted for 25.2 and 28.0%, respectively; double major curves were uncommon in both sexes. The anatomical distribution of curve location did not differ significantly between boys and girls (*p =* 0.16). Regarding curve severity, most cases were mild (10° – < 20°), followed by Cobb 20°–<30°, and curves ≥30° were relatively rare; no significant sex-based difference in Cobb angle categories was observed (*p =* 0.83).

When stratified by school level, primary school students showed a predominance of thoracic curves (84.0%), whereas middle school students had a more heterogeneous distribution across thoracic (43.8%), lumbar (23.4%), thoracolumbar (21.9%), and double major (10.9%) curves; the difference in curve location distribution was statistically significant (*p =* 0.001). With respect to severity, primary school cases were mainly mild (Cobb 10°–<20°: 76.0%), while middle school students had a higher proportion of moderate curves (20°–<30°: 43.8%) and a small proportion of curves ≥30° (8.8%); the difference in Cobb angle distribution by school level was statistically significant (*p =* 0.02) (Table 2).

**Table 2:** Comparison of scoliosis location and severity distribution among adolescents with adolescent idiopathic scoliosis by gender and grade.

Group	Total (*n*)	AIS (*n*, %)	*p-*value		Scoliosis location				Cobb angle			
				Thoracic	Lumbar	Thoracolumbar	Double major	*p-*value	10° – < 20°	20° – < 30°	30° – < 40°	*p-*value
Boys	2,718	55 (2.0)	<0.001	32 (58.2)	7 (12.7)	15 (27.3)	1 (1.8)	0.16*	30 (54.5)	21 (38.2)	4 (7.3)	0.83*
Girls	2,308	107 (4.6)		45 (42.1)	27 (25.2)	30 (28.0)	5 (4.7)		53 (49.5)	46 (43.0)	8 (7.5)	
Primary School (9–12 years)	1,228	25 (2.0)	0.01	21 (84.0)	1 (4.0)	2 (8.0)	1 (4.0)	0.001*	19 (76.0)	6 (24.0)	0 (0)	0.02*
Middle School (13–15 years)	3,798	137 (3.6)		60 (43.8)	32 (23.4)	30 (21.9)	15 (10.9)		73 (53.3)	52 (38.0)	12 (8.8)	
Grade												
Grade 4	638	9 (1.4)	0.011	6 (66.7)	0 (0.0)	3 (33.3)	0 (0.0)	0.001*	6 (66.7)	3 (33.3)	0 (0.0)	0.238*
Grade 5	590	16 (2.7)		15 (93.8)	1 (6.3)	0 (0.0)	0 (0.0)		13 (81.3)	3 (18.8)	0 (0.0)	
Grade 6	2,498	97 (3.9)		44 (45.4)	20 (20.6)	29 (29.9)	4 (4.1)		51 (52.6)	39 (40.2)	7 (7.2)	
Grade 7	1,300	40 (3.1)		17 (42.5)	13 (32.5)	8 (20.0)	2 (5.0)		22 (55.0)	13 (32.5)	5 (12.5)	

Values are presented as *n* (%). AIS (*n*, %) represents the crude radiographic detection rate within each subgroup. Percentages for scoliosis location and Cobb angle categories were calculated among radiographically confirmed AIS cases within each subgroup. Cobb angle categories were based on the maximum measured Cobb angle. Unmarked *p*-values were calculated using the chi-square test. *Fisher’s exact test.

### 3.2 Diagnostic performance of the AI-assisted structured-light screening workflow

Based on the embedded radiographic verification subset, which included all rescreen-positive students and a random sample of rescreen-negative students (*n* = 485), the unweighted diagnostic performance of the AI-assisted screening workflow was as follows: sensitivity, 0.988 (160/162); specificity, 0.923 (298/323); positive predictive value (PPV), 0.865 (160/185); negative predictive value (NPV), 0.993 (298/300); and overall accuracy, 0.944 (458/485), using radiographic confirmation with Cobb angle ≥10° as the reference standard. However, because only 300 of 4,841 screen-negative students underwent radiographic verification, these unweighted estimates were affected by partial verification. After IPW correction, the estimated numbers of false-negative and true-negative cases among screen-negative students were 32.3 and 4,808.7, respectively. The IPW-adjusted diagnostic performance was sensitivity, 0.832; specificity, 0.995; PPV, 0.865; NPV, 0.993; and overall accuracy, 0.989. These findings indicate that the AI-assisted screening workflow showed high specificity, PPV, NPV, and overall accuracy after correction for partial verification, although the corrected sensitivity was lower than the unweighted estimate.

### 3.3 Univariate analysis of factors associated with AIS

As shown in Table 3, factors related to posture, physical activity, lifestyle, psychological status, and body composition were significantly associated with AIS in both overall and gender-stratified groups.

**Table 3:** Univariate analysis of factors associated with AIS.

Independent variable	Category	Boys				Girls				Total			
		Number of participants (*n*)	Positive (*n*, %)	*χ* ^2^	*p-*value	Number of participants (*n*)	Positive (*n*, %)	*χ* ^2^	*p-*value	Number of participants (*n*)	Positive (*n*, %)	*χ* ^2^	*p-*value
BMI		2,718	55 (2.02)	—	0.814	2,308	107 (4.64)	—	0.175	5,026	162 (3.22)	—	0.026
Body density		2030	48 (2.36)	—	0.97	1,652	97 (5.87)	—	0.118	3,682	145 (3.94)	—	<0.001
WHR		1978	43 (2.17)	—	0.76	1,592	97 (6.09)	—	0.156	3,570	140 (3.92)	—	<0.001
Grip strength	Left	2,196	54 (2.46)	—	0.282	1804	106 (5.88)	—	0.509	4,000	160 (4.00)	—	0.238
	Right			—	0.397			—	0.813			—	0.363
	2*(Left − Right)/(Left + Right)			—	0.711			—	0.207			—	0.225
Thoracic mobility		2,206	55 (2.49)	—	0.985	1812	107 (5.91)	—	0.589	4,018	162 (4.03)	—	0.474
Is walking posture unnatural or unbalanced?	Never	2028	40 (1.97)	0.11	0.74	1764	69 (3.91)	9.35	0.002	3,792	109 (2.87)	6.19	0.013
	Sometimes	690	15 (2.17)			544	38 (6.99)			1,234	53 (4.29)		
Is the sitting posture proper?	Yes	410	8 (1.95)	0.013	0.91	406	6 (1.48)	11.683	<0.001	816	14 (1.72)	7.342	0.007
	No	2,308	47 (2.04)			1902	101 (5.31)			4,210	148 (3.52)		
Is standing posture abnormal?	Yes	730	21 (2.88)	3.16	0.075	534	31 (5.81)	1.91	0.167	1,264	52 (4.11)	4.06	0.044
	No	1988	34 (1.71)			1774	76 (4.28)			3,762	110 (2.92)		
Do you frequently engage in unilateral force-based sports?	Yes	1754	39 (2.22)	0.749	0.387	1,662	86 (5.17)	3.65	0.056	3,416	125 (3.66)	6.28	0.01
	No	964	16 (1.66)			646	21 (3.25)			1,610	37 (2.30)		
Frequency of physical exercise per week	More than 4 times	1,146	23 (2.01)	0.467	0.93*	830	29 (3.49)	8.169	0.04	1976	52 (2.63)	9.034	0.03
	3–4 times	684	15 (2.19)			680	42 (6.18)			1,364	57 (4.18)		
	1–2 times	772	14 (1.81)			662	27 (4.08)			1,434	41 (2.86)		
	Almost never exercise	116	3 (2.59)			136	9 (6.62)			252	12 (4.76)		
Is each exercise session ≥ 60 min?	Yes	518	8 (1.54)	0.757	0.384	232	7 (3.02)	1.607	0.205	750	15 (2.00)	4.374	0.036
	No	2,200	47 (2.14)			2076	100 (4.82)			4,276	147 (3.44)		
Level of fatigue after each exercise session	Quite tired (a lot of sweat)	724	12 (1.66)	7.90	0.019	284	11 (3.87)	1.15	0.562	1,008	23 (2.28)	4.99	0.083
	Slightly tired (some sweat)	1,338	21 (1.57)			1,206	61 (5.06)			2,544	82 (3.22)		
	Relaxed (mild sweat)	656	22 (3.35)			818	35 (4.28)			1,474	57 (3.87)		
MET	Value	2,352	50 (2.13)	—	0.364	2044	101 (4.94)	—	0.812	4,396	151 (3.43)	—	0.109
	≥4,500 MET·min/week			7.652	0.425			1.506	0.116			10.694	0.023
	High/medium/low			1.361	0.394			0.432	0.628			3.393	0.183
Backpack weight	Very light (≤1 kg)	44	0 (0.00)	1.089	0.78*	26	0 (0.00)	4.489	0.18*	70	0 (0.00)	4.9	0.18*
	Light (1–3 kg)	320	7 (2.19)			324	20 (6.17)			644	27 (4.19)		
	Moderate (3–5 kg)	1,466	31 (2.11)			1,290	52 (4.03)			2,756	83 (3.01)		
	Heavy (>5 kg)	888	17 (1.91)			668	35 (5.24)			1,556	52 (3.34)		
Habit of carrying a single-shoulder backpack	Never, always use both-shoulder backpack	2,176	42 (1.93)	2.051	0.56*	1772	78 (4.40)	4.694	0.2*	3,948	120 (3.04)	4.052	0.26*
	Occasionally use single-shoulder backpack	494	13 (2.63)			504	26 (5.16)			998	39 (3.91)		
	Often use single-shoulder backpack	34	0 (0.00)			20	1 (5.00)			54	1 (1.85)		
	Always use single-shoulder backpack	14	7 (50.00)			12	2 (16.67)			26	9 (34.62)		
Average daily study duration	Less than 8 h	766	20 (2.61)	2.436	0.49*	580	22 (3.79)	6.57	0.09	1,346	42 (3.12)	3.172	0.37
	9–10 h	1,236	22 (1.78)			1,006	59 (5.86)			2,242	81 (3.61)		
	11–12 h	480	10 (2.08)			530	20 (3.77)			1,010	30 (2.97)		
	12 h or more	236	3 (1.27)			192	6 (3.13)			428	9 (2.10)		
Desk and chair suitability	Completely suitable	930	18 (1.94)	1.813	0.61*	782	31 (3.96)	1.969	0.58*	1712	49 (2.86)	2.746	0.43*
	Fairly suitable	1,576	35 (2.22)			1,342	68 (5.07)			2,918	103 (3.53)		
	Slightly unsuitable	178	2 (1.12)			142	7 (4.93)			320	9 (2.81)		
	Completely unsuitable	34	0 (0.00)			42	1 (2.38)			76	1 (1.32)		
Family history	Yes	148	4 (2.70)	0.372	0.54*	158	7 (4.43)	1.156	0.28	306	14 (4.58)	1.975	0.16
	No	2,570	51 (1.98)			2,150	100 (4.65)			4,720	148 (3.14)		
Frequency of eating breakfast	Eat daily	2,290	45 (1.97)	2.372	0.5*	1864	84 (4.51)	1.816	0.61*	4,154	129 (3.11)	2.421	0.49*
	Occasionally skip	364	10 (2.75)			370	19 (5.14)			734	29 (3.95)		
	Frequently skip	54	0 (0.00)			58	4 (6.90)			112	4 (3.57)		
	Never eat	10	0 (0.00)			16	0 (0.00)			26	0 (0.00)		
Intake of milk and other dairy products	≥1 cup/day	1,478	31 (2.10)	2.106	0.55*	1,040	42 (4.04)	1.609	0.66	2,518	73 (2.90)	2.515	0.47
	4–6 cups/week	582	8 (1.37)			566	29 (5.12)			1,148	37 (3.22)		
	1–3 cups/week	484	11 (2.27)			544	28 (5.15)			1,028	39 (3.79)		
	None or <1 cup/week	174	5 (2.87)			158	8 (5.06)			332	13 (3.92)		
≥8 h of sleep per day	Yes	1,264	21 (1.66)	1.596	0.206	866	37 (4.27)	0.435	0.509	2,130	58 (2.72)	3.068	0.08
	No	1,454	34 (2.34)			1,442	70 (4.85)			2,896	104 (3.59)		
Is the quality of sleep satisfactory?	Yes	1988	35 (1.76)	0.156	0.693	1,246	42 (3.37)	13.042	<0.001	3,234	77 (2.38)	5.057	0.025
	No	730	20 (2.74)			1,062	65 (6.12)			1792	85 (4.74)		
Daily mental stress level	None	1,104	20 (1.81)	3.241	0.36*	860	37 (4.30)	4.335	0.23*	1964	57 (2.90)	4.663	0.20*
	A little	1,356	30 (2.21)			1,178	63 (5.35)			2,534	93 (3.67)		
	Moderate	188	2 (1.06)			188	5 (2.66)			376	7 (1.86)		
	High	70	3 (4.29)			82	2 (2.44)			152	5 (3.29)		
Stress relief ability	Fully able	968	14 (1.45)	2.899	0.41*	720	24 (3.33)	10.5	0.02*	1,688	38 (2.25)	12.569	0.006*
	Mostly able	1,250	29 (2.32)			1,070	63 (5.89)			2,320	92 (3.97)		
	Partially able	434	11 (2.53)			448	20 (4.46)			882	31 (3.51)		
	Not able at all	66	1 (1.52)			70	0 (0.00)			136	1 (0.74)		

Values are presented as number of participants and positive cases, *n* (%). Percentages represent crude detection rates within each category. *p*-values for continuous variables were calculated using independent-samples *t-*tests or non-parametric tests according to data distribution. For categorical variables, unmarked *p*-values were calculated using the chi-square test. *Fisher’s exact test or Fisher–Freeman–Halton exact test for sparse R × C tables.

For physical/metabolic indicators, BMI showed significance in the overall sample (*p =* 0.026), while waist-to-hip ratio and body density had stronger associations (*p <* 0.001), highlighting the link between body composition and spinal health.

Among posture-related factors, walking posture was significant overall (*p =* 0.04) and in girls (*p =* 0.002). Sitting posture showed differences in both the overall group (*p =* 0.007) and girls (*p <* 0.001), while standing posture was significant in the overall group (*p =* 0.04).

Regarding exercise, weekly frequency was significant in the overall group (*p =* 0.03) and girls (*p =* 0.04). Exercise duration ≥60 min was associated with AIS status (*p =* 0.036), as were MET ≥ 4,500 (*p =* 0.023). Frequent unilateral exertion showed significance overall (*p =* 0.01) and in girls (*p =* 0.043). Exercise fatigue was not significant (*p =* 0.08) but showed a non-linear trend.

Lastly, stress relief ability was inversely associated with AIS overall (*p =* 0.006) and in girls (*p =* 0.02), suggesting a protective role of emotional regulation (Table 4).

**Table 4:** Multivariable analysis of factors associated with screening-detected AIS.

Independent variable	Category	Boys (*n* = 1930)		Girls (*n* = 1,562)		Total (*n* = 3,492)	
		OR (95% CI)	*p*-value	OR (95% CI)	*p*-value	OR (95% CI)	*p*-value
Gender	Female					2.686 (1.806–3.996)	<0.01
	Male	1		1		1	
Grade	Grade 4	0.309 (0.064–1.483)	0.142	0.967 (0.355–2.637)	0.948	0.701 (0.309–1.590)	0.395
	Grade 5	0.913 (0.297–2.802)	0.873	1.787 (0.731–4.369)	0.203	1.494 (0.750–2.978)	0.253
	Grade 6	0.753 (0.355–1.593)	0.458	1.633 (0.887–3.006)	0.115	1.271 (0.796–2.031)	0.316
	Grade 7	1		1		1	
BMI		0.951 (0.871–1.038)	0.256	0.930 (0.861–1.004)	0.064	0.935 (0.884–0.989)	0.020
Is walking posture unnatural or unbalanced?	No	0.794 (0.224–2.818)	0.721	0.323 (0.136–0.767)	0.010	0.431 (0.219–0.849)	0.015
	Yes	1		1		1	
Is the standing posture abnormal?	The posture is less correct	0.913 (0.342–2.435)	0.856	1.229 (0.541–2.790)	0.622	1.127 (0.605–2.098)	0.707
	The posture is more correct	0.889 (0.380–2.082)	0.786	1.390 (0.693–2.790)	0.354	1.208 (0.709–2.057)	0.488
	The posture is completely correct	1		1		1	
Is the sitting posture proper?	No	0.780 (0.316–1.921)	0.589	3.215 (1.215–8.509)	0.019	1.808 (0.943–3.465)	0.074
	Yes	1		1		1	
Frequency of physical exercise per week	More than 4 times	0.774 (0.155–3.873)	0.755	0.998 (0.378–2.633)	0.997	0.914 (0.407–2.050)	0.827
	3–4 times	0.896 (0.394–2.039)	0.793	0.974 (0.517–1.837)	0.936	0.919 (0.561–1.505)	0.737
	1–2 times	1.055 (0.470–2.369)	0.896	1.651 (0.924–2.950)	0.090	1.422 (0.897–2.253)	0.134
	Almost never exercise	1		1		1	
Level of fatigue after each exercise session	Very relaxed (no sweat)	4.225 (1.02–5.245)	0.045	1.563 (0.460–5.312)	0.474	2.246 (0.885–5.699)	0.089
	Relaxed (a little sweat)	0.837 (0.302–2.316)	0.732	0.738 (0.321–1.696)	0.475	0.797 (0.428–1.487)	0.477
	A bit tired (a small amount of sweat)	1.258 (0.550–2.875)	0.587	1.053 (0.488–2.271)	0.896	1.139 (0.656–1.979)	0.644
	Quite tired (a large amount of sweat)	1		1		1	
≥4,500 MET·min/week	No	2.935 (0.361–23.836)	0.314	4.063 (0.527–31.322)	0.179	3.131 (0.738–13.280)	0.121
	Yes	1		1		1	
Do you frequently do single-sided exertion exercise?	Yes	2.245 (0.993–5.077)	0.052	1.605 (0.884–2.914)	0.120	1.797 (1.116–2.874)	0.016
	No	1		1		1	
Is each exercise session ≥ 60 min?	No	1.413 (0.512–3.897)	0.505	1.538 (0.618–3.828)	0.355	1.403 (0.721–2.733)	0.319
	Yes	1		1		1	
≥8 h of sleep per day	No	1.226 (0.619–2.428)	0.559	1.146 (0.694–1.892)	0.595	1.161 (0.780–1.729)	0.461
	Yes	1		1		1	
Is the quality of sleep satisfactory?	Yes	0.661 (0.293–1.491)	0.319	0.629 (0.365–1.083)	0.095	0.631 (0.404–0.985)	0.043
	No	1		1		1	
Do you experience daily psychological stress?	No	0.764 (0.218–2.674)	0.674	1.709 (0.605–4.828)	0.312	1.300 (0.590–2.865)	0.515
	Yes	1		1		1	

OR, odds ratio; CI, confidence interval. The dependent variable was screening-detected AIS status. ORs were estimated using complete-case multivariable binary logistic regression models. The boys and girls columns represent sex-stratified models, and the total column represents the overall model. The reference category is indicated by OR = 1. ORs were adjusted for the other variables included in the corresponding model. Variables were considered for inclusion according to the prespecified univariable screening criterion and model suitability.

### 3.4 Multivariable analysis of factors associated with screening-detected AIS

Multivariable binary logistic regression was performed to identify factors associated with screening-detected AIS, using AIS screening status as the dependent variable (0 = non-AIS, 1 = AIS). Variables with a *p* < 0.10 in the univariate analysis were entered into the multivariable model. A total of 3,492 adolescents (1,930 boys and 1,562 girls) with complete data on variables included in the multivariable analysis were included in the final models. The logistic regression analysis explored the associations between multiple factors and screening-detected AIS, with the results as follows:

In terms of physical and metabolic indicators, BMI was negatively associated with the odds of screening-detected AIS. The overall analysis showed that each unit increase in BMI was associated with lower odds of screening-detected AIS (OR = 0.935, 95% CI = 0.884–0.989, *p =* 0.020). Regarding sex, girls had significantly higher odds of AIS than boys (OR = 2.686, 95% CI = 1.806–3.996, *P*<0.01).

Regarding posture-related factors, correct walking posture was identified as a significant protective factor against AIS. Adolescents who reported a natural and balanced walking posture had significantly lower odds of AIS compared with those with imbalanced gait in the overall sample (OR = 0.431, 95% CI = 0.219–0.849, *p =* 0.015). This protective association was even more pronounced among girls (OR = 0.323, 95% CI = 0.136–0.767, *p =* 0.010). Additionally, incorrect sitting posture was associated with higher odds of screening-detected AIS in girls, with an OR of 3.215 (95% CI = 1.215–8.509, *p =* 0.019).

In terms of exercise behavior, frequent engagement in one-sided exertion sports was associated with higher odds of screening-detected AIS, with an OR of 1.797 (95% CI = 1.116–2.874, *p =* 0.016). Regarding post-exercise fatigue, boys who felt “relaxed (slightly sweating)” after exercise had significantly higher odds compared to those who felt “heavily fatigued (profuse sweating),” with an OR of 4.225 (95% CI = 1.02–5.245, *p =* 0.045).

In lifestyle and psychological factors, good sleep quality was associated with lower odds of screening-detected AIS. In the overall sample, individuals with good sleep quality had a significantly lower odds compared to those with poor sleep quality (OR = 0.631, 95% CI = 0.404–0.985, *p =* 0.043). However, other psychological variables such as adequate sleep duration (≥8 h) and presence of mental stress showed no significant associations (*p* > 0.05).

## 4 Discussion

This large-scale school-based screening conducted in Shanghai, a representative megacity in China, systematically evaluated the epidemiological characteristics and factors associated with AIS among primary and secondary school students. The primary novelty of this study lies in the large-scale, real-world implementation of an AI-assisted structured-light screening workflow within the school health system of a major Chinese metropolis, a context that has been rarely examined in the existing literature. In addition, the study integrates AI-based surface morphological screening with a comprehensive epidemiological assessment of factors associated with AIS. The findings revealed a crude radiographically confirmed AIS detection rate of 3.2%. After IPW correction for partial verification of screen-negative students, the estimated population prevalence was 3.8%. Compared with other regions in China, the IPW-adjusted prevalence in Shanghai falls within the mid-range. Eastern coastal provinces typically report confirmed rates between 2 and 3% (35–37), whereas some central and western provinces report suspected prevalence as high as 8%. Internationally, the adjusted prevalence in this study is close to the lower bound of that reported in Europe and North America (approximately 2%), lower than the average range (2–4%), and significantly lower than high-risk populations, where prevalence may reach 6–10% (38, 39).

Methodologically, this study implemented a large-scale, real-world application of a non-contact AI-assisted structured-light 3D imaging system within a megacity school health setting, providing more standardized and objective surface assessment and reducing examiner-related variability inherent to traditional visual or angle-ruler–based screening (40–42). Because of concerns regarding radiation exposure, cost, and logistical feasibility, relatively few school-based screening studies have incorporated radiography as a reference standard (43). In contrast, without universal radiography, we embedded radiographic confirmation into the workflow: with informed consent on a voluntary basis, screen-positive students were referred for X-ray verification, and a random sample of screen-negative students was invited for radiography to assess potential false negatives. This design improved the reliability of case ascertainment and the robustness of prevalence estimation, enhancing interpretability and cross-study comparability. Supported by acceptable diagnostic performance in the embedded verification analysis, the AI-assisted system may be integrated into routine school health surveillance and offers a practical pathway toward standardized screening in megacities.

In terms of associated-factor analysis, this study constructed a relatively comprehensive profile of factors associated with screening-detected AIS. Lower BMI was associated with significantly higher odds, supporting the hypothesis that low body mass or inadequate muscle mass may compromise spinal stability, consistent with findings by Scaturro and Burwell (44, 45). Postural habits, including abnormal gait and poor sitting posture, were also identified as independent factors associated with AIS, with risk increasing proportionally with the frequency of postural deviation. Among students in megacities, prolonged sedentary study and excessive use of electronic devices may contribute to these issues. Prior research has shown that gait abnormalities can induce pelvic rotation and muscle imbalance, subsequently altering spinal load distribution and axis alignment (46, 47), while prolonged forward-leaning posture during desk work can concentrate stress in the thoracolumbar region, potentially triggering structural changes (48–50).

Regarding physical activity, both low-intensity exercise and unilateral strength-dominant sports were significantly associated with higher odds of screening-detected AIS. These results are consistent with findings by Jeon and others among sport-specific youth populations (51), suggesting that asymmetric movement patterns may have lasting effects on spinal biomechanics (52). For adolescents in megacities, academic pressures and space limitations further reduce access to effective exercise, potentially exacerbating the negative impact of inadequate physical activity on spinal health (53).

In the domain of lifestyle factors, poor sleep quality emerged as an independent factor associated with screening-detected AIS, particularly pronounced among female students. Potential mechanisms may involve disruption of growth hormone secretion, dysregulation of the nervous system, and reduced muscle support function (54). Given the widespread prevalence of irregular sleep patterns among adolescents in megacities, these findings carry important public health implications.

Beyond individual behavioral factors, these associations should also be viewed within the broader lifestyle context of rapidly urbanizing megacities such as Shanghai. The accelerated pace of urbanization has reshaped adolescent daily routines toward prolonged sedentary study, intensive digital device use, and increasingly indoor-centered living patterns (55, 56). Such environments reinforce sustained static sitting and forward-leaning postures, which may amplify the adverse impact of improper sitting posture and gait imbalance on spinal development, as suggested by previous biomechanical studies (49).

Furthermore, high-density urban settings often limit access to diverse physical activity spaces. As a result, adolescents are more likely to engage in exercise patterns that are either insufficient in overall intensity (57, 58) or concentrated in unilateral, skill-specific activities. These patterns may exacerbate muscular imbalance during growth and align with findings from prior research (59).

Sleep disturbances are also more prevalent in megacities due to academic pressure, long commuting distances, and extensive evening screen exposure. Such disruptions in sleep may impair neuromuscular recovery and postural control, potentially increasing susceptibility to spinal imbalance (60, 61). Taken together, these urban lifestyle dynamics indicate that environmental constraints and behavioral exposures interact in ways that create a heightened-risk setting for adolescent spinal health in megacities.

This study introduces an innovative, scalable screening model in a megacity context, enhancing the generalizability of adolescent spinal health data. It is the first to report suspected and confirmed rates in parallel, improving academic clarity and international comparability. The validated use of AI-assisted 3D imaging offers a precise, safe method for large-scale surveillance. By integrating physical, functional, and behavioral variables, the study establishes a multidimensional risk framework to support targeted prevention and intervention strategies.

This study has several limitations. Owing to its cross-sectional design, the observed associations between potential factors and AIS reflect correlation rather than causation, and neither temporal sequence nor causal direction can be established. The sample consisted of students aged 9–15 years from six schools across five districts in Shanghai; although stratified cluster sampling was applied, the findings may still be shaped by school participation, grade composition, and urban lifestyle characteristics, so generalization to other cities or non-urban areas should be made with caution. Several key variables were collected primarily through self-reported questionnaires, including postural habits, exercise intensity, participation in unilateral sports, sleep, and psychological stress, which may introduce recall bias and social desirability bias. Radiographic verification was not conducted for all screening-negative participants; instead, 300 randomly sampled screen-negative students underwent X-ray re-examination, and inverse probability weighting was used to reduce verification bias. However, residual misclassification and estimation uncertainty may persist because only 2 AIS cases were identified in the verified screen-negative sample, and the IPW-adjusted estimate depends on the assumption that the verified screen-negative students were representative of all screen-negative students. Although the AI-based structured-light 3D screening system showed good diagnostic agreement in real school settings, its performance may vary with device models, operator standardization, ambient lighting, participant compliance with standing posture, and algorithm updates, underscoring the need for local revalidation and continuous quality control before wider implementation. Despite adjustment for multiple domains such as body morphology, physical fitness, and lifestyle factors, unmeasured or insufficiently measured confounders may remain, including pubertal stage, genetic background, bone density or hormone-related indicators, and more granular assessments of sedentary behavior and screen exposure. Future multicenter longitudinal studies incorporating more objective behavioral measurements are warranted to strengthen the evidence base.

## 5 Conclusion

AIS was more frequently detected in girls and in middle school students, and was associated with lower BMI, imbalanced gait, improper sitting posture, insufficient or unilateral exercise, and poor sleep quality, with posture- and exertion-related patterns particularly evident in girls. The AI-assisted structured-light 3D back-scanning system showed acceptable diagnostic performance against radiographic Cobb angle and can serve as a non-contact adjunct in school-based screening. These findings highlight the need to combine AI-assisted postural assessment with targeted health education and behavior-focused interventions to help protect adolescent spinal health in urban settings.

## Data Availability

The original contributions presented in the study are included in the article/supplementary material, further inquiries can be directed to the corresponding authors.
